# Is it premature to formulate recommendations for policy and practice, based on culture and health research? A robust critique of the CultureForHealth (2022) report

**DOI:** 10.3389/fpubh.2024.1414070

**Published:** 2024-07-11

**Authors:** Mette Kaasgaard, Katarzyna Grebosz-Haring, Christina Davies, George Musgrave, Jahnusha Shriraam, J. Matt McCrary, Stephen Clift

**Affiliations:** ^1^Pulmonary Research Unit (PLUZ), Department of Medicine, Zealand University Hospital, Naestved, Denmark; ^2^Department of Regional Health Research, Faculty of Health Sciences, University of Southern Denmark, Odense, Denmark; ^3^Interuniversity Organisation Science and Art, Paris Lodron University Salzburg, Mozarteum University Salzburg, Salzburg, Austria; ^4^Department of Art History, Musicology and Dance Studies, Paris Lodron University Salzburg, Salzburg, Austria; ^5^Centre for Arts, Mental Health and Wellbeing, School of Allied Health and School of Humanities, The University of Western Australia, Perth, WA, Australia; ^6^Institute for Creative and Cultural Entrepreneurship (ICCE), Goldsmiths, University of London, London, United Kingdom; ^7^Music and Health Research Institute (MHRI), University of Ottawa, Ottawa, ON, Canada; ^8^Department of Human Genetics, Hannover Medical School, Hanover, Germany; ^9^Prince of Wales Clinical School, University of New South Wales, Kensington, NSW, Australia; ^10^Sidney De Haan Research Centre for Arts and Health, Canterbury Christ Church University, Canterbury, United Kingdom; ^11^International Centre for Community Music, York St John University, York, United Kingdom

**Keywords:** culture, arts and health, scoping reviews, evidence, health policy

## Abstract

**Introduction:**

Arts and health practice and research has expanded rapidly since the turn of the millennium. A World Health Organization scoping review of a large body of evidence claims positive health benefits from arts participation and makes recommendations for policy and implementation of arts for health initiatives. A more recent scoping review (CultureForHealth) also claims that current evidence is sufficient to form recommendations for policy and practice. However, scoping reviews of arts and health research—without critical appraisal of included studies—do not provide a sound basis for recommendations on the wider implantation of healthcare interventions.

**Methods:**

We performed a detailed assessment of 18 Randomised Controlled Trials (RCTs) on arts-based interventions included in Section 1 of the CultureForHealth report using the Joanna Briggs Institute Critical Appraisal Tool for RCTs (2023).

**Results:**

The 18 RCTs included demonstrated considerable risks of bias regarding internal and statistical conclusion validity. Moreover, the trials are substantially heterogeneous with respect to settings, health-issues, interventions, and outcomes, which limits their external validity, reliability, and generalisability.

**Conclusions:**

The absence of a critical appraisal of studies included in the CultureForHealth report leads to an overinterpretation and overstatement of the health outcomes of arts-based interventions. As such, the CultureForHealth review is not a suitable foundation for policy recommendations, nor for formulating guidance on implementation of arts-based interventions for health.

## Summary

The CultureForHealth report (1) maps the literature in the field of culture, well-being and health in order to inform policy recommendations for Europe. The scoping review methodology employed did not include critical screening of the research studies included. In this paper, we report a critical assessment of 18 randomised controlled trials (RCTs) cited in Section 1 on “Culture and Health” of the CultureForHealth report using the Joanna Briggs Institute (JBI) Critical Appraisal Tool for RCTs (2023). The appraisals reveal considerable risks of bias across all trials, which limit their internal and statistical conclusion validity. The absence of a critical appraisal of studies included in the CultureForHealth report leads to an overinterpretation and overstatement of the health outcomes of arts-based interventions. As such, the CultureForHealth review is not a suitable foundation for policy recommendations, nor for formulating guidance on implementation of arts-based interventions for health.

## Introduction

Since the turn of the millennium, there has been increasing attention internationally towards the potential wellbeing and health benefits of engagement with culture and creative arts activities. This interest has been motivated by the perceived need to draw on community assets outside the traditional medical field to address growing challenges in population health and demands made on healthcare systems due to funding constraints. Moreover, there is increasing recognition that medical science may face limitations in dealing with progressive long-term conditions and health inequalities and that greater efforts are needed to address social determinants of health across the life course (2). Thus, engagement with culture and creative arts are suggested as potential resources to support health through prevention, promotion, care, and treatment (3).

Efforts have been made to review the growing international body of research on culture, arts, and health in scoping and narrative evidence reviews, notably, by the All-Party Parliamentary Group for Arts, Wellbeing and Health, UK (2017, 2023) (4, 5) and in reports reviewing evidence, e.g., from Europe (5–10), the US (11), and Australia (12). One considerable boost to further developments in the field has been given by a scoping review published by the World Health Organization (WHO) in 2019, summarising findings from over 3,000 studies (13). In addition, the WHO has supported the establishment of collaborating centres for arts and health research at University College London (UCL), and additional centres in the Steinhardt School at New York University and Edgehill University (UK). In 2023, the WHO and the Jameel Arts & Health Lab (New York, USA) announced a special Lancet Global Series on the health benefits of the arts (14–16) which “will show the scientific basis of the arts' role in health with rigour, and help position artists and scientists as necessary partners towards health and wellbeing for all” (16).

In 2022, Culture Action Europe (17) published a further scoping review - the CultureForHealth report (1) — to support: “Bottom-Up Policy Development for Culture & Wellbeing in the EU” (17). The report aimed “to synthesise existing evidence on the positive effect of arts and cultural activities on health and wellbeing” (17) and “to inform policy recommendations for Europe” (p. 24) (1). Studies published between 2005 and November 2021 were identified for the review following a search strategy using PubMed, Scopus, and other sources (see p. 26). The authors of the CultureForHealth report acknowledge limitations with their search strategy and that “our search terms may not have covered all possible valuable aspects of our focus theme very accurately” (p. 25). Section 1 of four sections in the report focuses on health benefits from cultural and arts participation and includes details of 137 empirical studies (including controlled trials and quasi-experimental, observational, qualitative, and mixed methods studies) and reviews (including systematic, scoping, and narrative reviews). Sections 2 to 4 are concerned with culture and subjective wellbeing, community wellbeing, and COVID-19, and are not considered in this paper.

The CultureForHealth report also includes a large section describing key challenges to public health across Europe and the authors state (p. 5) (1) that culture could help to effectively tackle these challenges:

1. The need for an increased focus on health promotion and disease prevention.
2. A growing mental health crisis.
3. The need to support the broader health and wellbeing of young people.
4. Ongoing changes to the labour markets, patterns of work and the economy.
5. An ageing population.
6. The association between ill health and patterns of inequality.
7. The need to promote active citizenship.
8. The mental health challenges faced by forcibly displaced people.

The findings and emerging initiatives associated with the CultureForHealth project have been showcased at various events (see weblinks in the Appendix p. 2), a guide tailored for practitioners has been published (18), and several activities are already in the process of implementation (see weblinks in the Appendix p. 1).

The WHO and the CultureForHealth scoping reviews, and other reports, do summarise a large body of empirical evidence on the benefits of arts initiatives, especially music and dance. However, scoping reviews alone are generally not a satisfactory basis for recommending healthcare interventions (19–21). Accordingly, serious concerns have been raised regarding the limitations of the WHO report (22, 23) for its lack of critical appraisal of the studies included and for its willingness to take conclusions drawn in primary research studies at face value. Further critical papers have stressed the need for treating findings and conclusions from research and evidence reviews within arts and health with considerable caution until strong evidence has been established (22, 24–28).

In this paper, we present the results of a critical appraisal of RCTs included in Section 1 in the CultureForHealth report. We focus on RCTs as these are widely regarded as providing the most robust source of evidence on effects of interventions on health outcomes and are, moreover, central to systematic critical reviews and acknowledged frameworks for clinical guidelines for safe, effective, and evidence-based healthcare interventions (29–33). In addition, we analyse the impact of this appraisal on the validity of the report conclusions and policy recommendations.

## Methods

### Inclusion and analysis procedure

Between February and April 2023, we identified all RCTs on arts/cultural interventions included in Section 1 of the CultureForHealth report (1). Table 2 in the report lists 20 RCTs, but one of the sources identified as such is not a randomised trial, and a second is not a trial report. This leaves 18 trials and data extraction was undertaken to describe characteristics of these RCTs. The RCTs were concerned primarily with singing, dance, and music listening interventions. We then undertook an appraisal of the RCTs using the revised JBI tool to assess risk of bias in RCTs (34) with respect to internal and statistical conclusion validity. The tool consists of thirteen questions with associated guidance (see Table 1), based on the Joanna Briggs Critical Appraisal Tool for the Assessment of Risk of Bias for Randomised Controlled Trials (34).

**Table 1:** JBI critical appraisal tool for the assessment of risk of bias for RCTs (34): questions and guidance.

**Question**	**Domain and potential bias**	Level of appraisal^*^	**Guidance on what reviewers should cheque**
1: Was true randomisation used for assignment of participants to treatment groups?	Selection and allocation	Study	Was a true chance (random) procedure used? For example, was a list of random numbers used? Was a computer-generated list of random numbers used? Was a statistician, external to the research team, consulted for the randomisation sequence generation?
2: Was allocation to groups concealed?	Selection and allocation	Study	Was an appropriate allocation concealment procedure used? For example, was central randomisation used? Were sequentially numbered, opaque, and sealed envelopes used?
3: Were treatment groups similar at the baseline?	Selection and allocation	Study	Are the participants from the compared groups similar with regard to the characteristics that may explain the effect? NB: Do not only consider the *P* value for testing of differences.
4: Were participants blind to treatment assignment?	Administration of intervention	Study	Was an appropriate blinding procedure used? Were participants aware of the treatment arm they were allocated to?
5: Were those delivering the treatment blind to treatment assignment?	Administration of intervention	Study	Were those delivering the treatment unaware of the assignments of participants to the compared groups?
6: Were treatment groups treated identically other than the intervention of interest?	Administration of intervention	Study	Are there other exposures or treatments occurring at the same time as the cause? Is it plausible that the effect may be explained by other exposures or treatments occurring at the same time as the cause?
7: Were outcome assessors blind to treatment assignment?	Assessment of outcomes	Outcome	Were those assessing the treatment's effects on outcomes unaware of the assignments of participants to the compared groups?
8: Were outcomes measured in the same way for treatment groups?	Assessment of outcomes	Outcome	Was the same instrument or scale used? Was the measurement timing the same? Were the measurement procedures and instructions the same?
9: Were outcomes measured in a reliable way?	Assessment of outcomes	Outcome	NB: This question is about the reliability of the measurement performed in the study, and not about the validity of the measurement instruments/scales used in the study.
10: Was follow-up complete and, if not, were differences between groups in terms of their follow-up adequately described and analysed?	Participant retention	Result	Was follow-up complete? If there are differences between groups with regard to the loss to follow-up (numbers/proportions and reasons), was there an analysis of patterns of loss to follow-up? NB: Question 10 is not about intention-to-treat [ITT] analysis. This is covered by Q11.
11: Were participants analysed in the groups to which they were randomised?	Statistical conclusion validity	Result	Were participants analysed in the groups to which they were initially randomised, regardless of whether they participated in those groups and regardless of whether they received the planned interventions (ITT analysis)? NB: The ITT analysis is a type of statistical analysis recommended in the Consolidated Standards of Reporting Trials (CONSORT) statement on best practices in trials reporting, and it is considered a marker of good methodological quality of the analysis of results of a randomised trial.
12: Was appropriate statistical analysis used?	Statistical conclusion validity	Result	Reviewers should cheque the following aspects: if the assumptions of the statistical tests were respected; if appropriate statistical power analysis was performed; if appropriate effect sizes were used; if appropriate statistical methods were used given the nature of the data and the objectives of statistical analysis (e.g., association between variables, prediction, survival analysis).
13: Was the trial design appropriate?	Statistical conclusion validity	Study	Was standard RCT design (individual randomisation, parallel groups) adopted. If not, what form of trial was conducted and what rationale is offered?

^*^In the 2023 revision of the JBI Appraisal Tool for the Assessment of Risk of Bias for Randomised Controlled Trials (RCTs), Questions 1–6 and 13 operate at the study level. Questions 7–9 relate to outcomes assessed and so mixed assessments are possible. Questions 10–12 are addressed in relation to each outcome result. In the current evaluation, however, RCTs were appraised at a study level for questions 10–12.

To ensure accurate appraisals, a two-stage strategy was employed:

***Stage 1:*** Between July 2023 and November 2023, each trial report was appraised independently by two members of the research team for each domain (singing: MK, SC; dance: KG-H, SC; music listening and games: JS, SE). The assessors then met online and discussed their ratings and, where differences of opinion had arisen, an agreed judgment was reached through discussion and re-reading of the papers. Where two team members did not resolve differences, another member of the team was involved as moderator (JMM).

***Stage 2:*** In addition, in December 2023, all papers were read again and appraised against each JBI question (34) by a single team member (SC), based on appropriate data extraction from all papers (Supplementary Table 1). This helped to ensure that a reasonable relativity in judgement could be achieved, as assessments were made against the same standards. These judgements were then independently scrutinised by two other team members (MK, KHG) and any difference of opinion discussed and resolved. Findings from the second strategy were then used to moderate agreed ratings arising from the first strategy.

## Results

### Characteristics of the RCTs

Section 1 of the CultureForHealth report includes reference to 18 RCTs (see Table 2). The art-form investigated varied: nine of the 18 trials were on group singing (35–43); five trials examined group dancing (44–48); three trials involved a musical intervention (49–51); and the final trial examined effects of games and painting (52). Moreover, the control arm(s) varied substantially from other arts-/culture-based activities to no intervention, or usual care (standard, health-care-based treatment).

**Table 2:** Characteristics of the 18 RCTs cited in Section 1 of the CultureForHealth Report (1).

**RCTs included**	**References**	**Country**	**Target group**	**Intervention and control**	**Outcome measure(s)**	**Participants randomised**
**RCTs on group singing**						
Bonilha et al.	(36)	Brazil	People with Chronic Obstructive Lung Disease (COPD)	Group singing vs. Handcraft	Maximal respiratory pressures, spirometry measures, breathlessness and quality of life (SGRQ)	43
Coulton et al.	(35)	UK	Older people 60+ years	Group singing vs. no intervention	Mental health-related quality of life (SF-12), anxiety and depression (HADS), quality of life (EQ-5D)	258
Fancourt and Perkins	(37)	UK	Mothers with symptoms of post-natal depression	Group singing vs. group play vs. usual treatment	Depression (EPDS)	153
Feng et al.	(38)	Singapore	Older people with early cognitive impairment	Group singing vs. health education	Composite measure of cognitive function (CCTS)	93
Ganzoni et al.	(39)	Switzerland	Patients with acquired or congenital structural heart disease	Group singing and breathing exercises vs. usual treatment	Maximal respiratory pressures and quality of life (MHLFQ)	22
Liu et al.	(40)	China	Patients with Chronic Obstructive Lung Disease (COPD) and mild depression	Group singing vs. health education	Depression (HADS) and quality of life (CCQ)	60
Philip et al.	(41)	UK	Patients with Chronic Obstructive Lung Disease (COPD)	Group singing in person and online vs. usual treatment	SF-36 physical and mental components, balance confidence (ABC scale), anxiety (GAD-7), depression (PHQ-9), COPD quality of life (CAT), breathlessness (MRC)	18
Pongan et al.	(42)	France	Patients with mild cognitive disorder 60+ years	Group singing vs. painting	Three measures of chronic pain (NRS, SVS, BPI), anxiety (STAI), depression (GDS), quality of life (EQ-5D)	65
Wulff et al.	(43)	Germany	Mothers with post-partum depression	Group singing vs. usual treatment	Depression (EPDS), bonding (PBQ), anxiety (STAI)	120
**RCTs on group dancing**						
Cruz-Ferreira et al.	(44)	Portugal	Older women 65+ years of age	Group creative dance vs. no intervention	Composite measure of physical fitness (SFT)	68
Duncan et al.	(45)	USA	Patients with clinically defined Parkinson's	Community Argentine Tango vs. usual treatment	Parkinson's severity (MDS-UPDRS), balance (MiniBESTest), gait (FOG-Q), upper extremity function (9HPT), distance walked in 6 min 6MWT	62
Lazarou et al.	(46)	Portugal	Elders 55–75 years with mild cognitive impairment	International ballroom dancing vs. no intervention	Multiple tests of cognitive function and mood and depression (e.g., MMSE, RBMT, BDI, PSS, RAVLT)	154
Kalsatou et al.	(47)	Greece	Patients with schizophrenia	Greek traditional dancing vs. usual treatment	Multiple tests of functional capacity (e.g., 6MWT, SST, BBS), mental assessment and quality of life (GAFS, QLESQ)	31
Marquez et al.	(48)	USA	Late middle-aged and older Latinos 55+ years	Latin dancing vs. health education	Multiple tests of physical health (e.g., BMI, blood pressure), cognitive function (e.g., TMT, DST)	57
**RCTs on music**						
Caprilli et al.	(49)	Italy	Children 4–13 years undergoing venipuncture	Live music and parental support vs. parental support	Observation of child distress (OSBD-A), child self-reported pain (VAS with six faces)	108
Huang et al.	(50)	China	Healthy medical students receiving orthodontic treatment	Customised brainwave music vs. CBT vs. no intervention	Self-reported pain (VAS), multiple measures of brain electrical activity	36
Qin et al.	(51)	China	Hospital patients with ankylosing spondylitis	Personalised Chinese music vs. painting vs. usual treatment	Quality of life (GQO-LI-&4)	120
**RCTs on games and painting**						
Forouzandeh et al.	(52)	Iran	Children 3–12 years undergoing surgery	Interactive games vs. painting vs. usual treatment	Observation of child anxiety (mYPAS)	172

6MWT, 6-Min Walk Test; 9HPT, Nine-Hole Peg Test; ABC, Activity-specific Balance Confidence scale; BBS, Berg Balance Scale; BDI, Beck Depression Inventory; BMI, Body Mass Index; BPI, Brief Pain Inventory; CAT, COPD assessment Test; CCQ, Clinical COPD Questionnaire; CCTS, Composite Cognitive Test Score; DST, Digit Span Test; EPDS, Edinburgh Postnatal Depression Scale; EQ-5D, EuroQoL Five Dimensions scale; FOG-Q, Freezing of Gait Questionnaire; GAD-7, Generalised Anxiety Disorder scale; GAFS, Global Assessment of Functioning Scale; GDS, Geriatric Depression Scale; GQO-LI-74, Generic Quality of Life Inventory; HADS, Hospital Anxiety and Depression Scale; MDS-UPDRS, composite measure of function in Parkinson's; MHLFQ, Minnesota Living with Heart Failure Questionnaire; MiniBESTest, a measure of balance in Parkinson's; MMSE, Mini Mental State Examination; MRC, Medical Research Council breathlessness scale; mYPAS, Modified Yale Preoperative Anxiety Scale; NRS, Numerical Rating Scale; OSBD-A, Amended Form of the Observational Scale of Behavioural Distress; PBQ, Postpartum Bonding Questionnaire; PHQ-9, Patient Health Questionnaire scale; PSS, Perceived Stress Scale; QLESQ, Quality of Life Enjoyment and Satisfaction Scale; RAVLT, Rey Auditory Verbal Learning Test; RBMT, Rivermead Behavioural Memory Test; SF-12, York Short Form 12 Health Survey; SF-36, Short Form 36 Health Survey; SFT, Senior Fitness Test; SGRQ, St George's Respiratory Questionnaire; SST, Sit to Stand Test; STAI, State Trait Anxiety Inventory; SVS, Subjective Visual Scale; TMT, Trail Making Test; VAS, Visual Analogue Scale.

The trials were conducted in 12 different countries: UK: 3 (35, 37, 41), China: 3 (40, 50, 51), Greece: 2 (46, 47), the USA: 2 (45, 48), and one each in Brazil (36), France (42), Germany (43), Iran (52), Italy (49), Portugal (44), Singapore (38), and Switzerland (39), i.e., only nine trials were conducted within the European Union. There was substantial heterogeneity regarding study durations, settings, health issues, participants, and outcome measure, and the RCTs varied considerably in size [smallest study (41): eighteen participants; largest (35): 258 participants]. Only five trials reported a prospective power calculation with target sample sizes achieved. The rest were either under-powered or did not report a prospective power calculation. In addition, nine trials were explicitly described as pilot studies (35, 39, 41, 48), as a “pioneer” study (36), or as “exploratory” studies (37, 40, 43, 46). Only three trials (35, 39, 41) reported on achievement of minimal clinically important differences (MCID) related to study outcomes and study findings observed.

### Pre-registration, ethics, and CONSORT

Eleven trials provide details of pre-registration, including the trials' register number, so that the protocol is accessible. However, for seven trials, there is no indication that the study was pre-registered. These include two trials conducted in China (40, 51), both studies in Greece (46, 47), one in the USA (48), and those in Italy (49) and Portugal (44). All trials apart from one (51) report ethical approval, however, only seven provide an ethics committee reference number. All reports indicate that participants gave informed consent. Seven make explicit reference to CONSORT guidelines (53) and report a standard CONSORT flow diagram. However, only one includes a CONSORT checklist (39). Two trials refer to CONSORT guidelines, but the flow diagram is either incomplete (46) or is non-standard (43). Five trial reports (40, 42, 48, 50, 52) do not explicitly refer to CONSORT but do include a participant flow chart (42, 44, 47, 50, 52). Finally, two reports (46, 51) make no reference to CONSORT and do not include any participant flow diagram.

Notably, authors in all trial reports acknowledge substantial limitations to their study and recommend further research with the conduct of large-scale trials.

### Assessment of the 18 RCTs using the JBI Critical Appraisal Tool (2023)

Table 3 reports the consensus JBI tool (34) assessments of each of the 18 RCTs. Supplementary Table 1 provides example quotations from the trial reports to clarify the variations in ratings of the RCTs for questions in the JBI tool.

**Table 3:** Assessments of 18 RCTs cited in Section 1 of the CultureForHealth Report (1) using the JBI assessment tool (34).

**Item**	**1**	**2**	**3**	**4**	**5**	**6**	**7**		**8**	**9**		**10**	**11**	**12**	**13**
**RCTs**	Truly randomised	Allocation concealed	Similar at baseline	Participants blind to treatment	Deliverers blind to treatment	Groups treated identically	Outcome assessors blind^*^		Outcomes measured same way	Outcome measure(s) Reliable		Follow-up complete	Participants analysed as allocated	Statistical analysis appropriate	Design appropriate
Bonilha et al. (36)															
Coulton et al. (35)^a,b^															
Fancourt and Perkins (37)															
Feng et al. (38)^c^															
Ganzoni et al. (39)^b^															
Liu et al. (40)															
Philip et al. (41)^a^															
Pongan et al. (42)^a,b^															
Wulff et al. (43)^c^															
Cruz-Ferreira et al. (44)															
Duncan et al. (45)^a,b^															
Kalsatou et al. (47)															
Lazarou et al. (46)															
Marquez et al. (48)															
Caprilli et al. (49)			N/A												
Huang et al. (50)															
Qin et al. (51)															
Forouzandeh et al. (52)															

Red = No, Yellow = Unclear, Green = Yes, Q3, N/A = no baseline assessment. ^*^Patient-reported outcomes not blind.^a^Q11, Intention-to-treat analysis undertaken;^b^Q12, Prospective power calculation/sample achieved;^c^Q12, Prospective power calculation/sample not achieved.

### JBI questions with high ratings

Table 3 shows that all trials were rated positively with respect to three questions: Q6: in all trials, treatment groups were treated identically apart from the intervention(s) of interest; Q8: in all trials, outcomes were measured in the same way; and Q13: for all trials, a standard and appropriate parallel groups design was employed. In two cases, however, the trials involved three arms [Fancourt and Perkins (37)—intervention, active control and treatment as usual; Qin (51)—music listening, painting and usual treatment]. All other trials involved two-arm intervention-control designs.

### JBI questions with low ratings

All trials were rated as being at high risk of bias for two criteria: Q4: for all trials, participants were not blinded to the conditions they were allocated to, and Q5: for all trials, deliverers of interventions were not blinded to the activity (e.g., singing or dancing).

### JBI questions with varied ratings

For the remaining questions in the JBI tool, assessments varied:

Q1: Seven trials (35, 38, 39, 41, 44, 46, 48, 50) provide sufficient information that a satisfactory randomisation procedure was followed. In the remainder, we judged this to be unclear. Given, however, that all studies are described as RCTs, it was not possible to conclude that true randomisation did not take place for any study.
Q2: Concealment was judged to have taken place where a satisfactory randomisation process was described. Otherwise, concealment was unclear in most cases. For two trials, however, the authors explicitly state that concealment of allocation from researchers did not happen (37, 43).
Q3: For 12 trials, trial groups appeared to be equivalent at baseline, with no significant differences on any outcome measure reported. For five trials, however, especially where sample sizes were small, marked differences in outcome means were apparent, even though these were reported as not statistically significant.
Q7: On the blinding of outcome assessors, Yes/No ratings were given for 11 of the 18 trials because multiple outcomes were assessed. Where the outcomes were objective assessments (e.g., lung function, cognitive function), the assessors were blinded. However, where outcomes were participant reported (e.g., quality of life, ratings of risk of developing depression), the assessments were not blind. For six trials, single or multiple outcomes were patient reported and so “No” rating is given on blinding of assessment at study level. In only two trials (49, 52) were outcome assessments satisfactorily blinded.
Q9: In two trials (49, 52), information was provided on the reliability of the data reported. These are both studies of children undergoing medical procedures where an observer assessed their levels of anxiety/fear. For a further study (44), reliability estimates for one of the outcomes assessed was presented but not for all of them. In all other trials, there is no direct evidence that the data reported was reliable. In most cases, however, it is clear that previously validated assessment procedures or questionnaires and scales were employed.
Q10: For 14 of the 18 trials, follow-up of participants throughout the trial was complete with very little or no attrition. For four trials (35, 36, 46, 48), however, attrition was 15% or greater, and so judged as substantial.
Q11: In most trials, participants were judged to have been analysed as initially randomised, either because an intention-to-treat analysis was explicitly undertaken, or because there was little or no attrition in the course of the study. For three studies (36, 46, 48), however, this was not the case.
Q12: For five trials (35, 39, 42, 45, 49), the statistical analysis reported was judged to be appropriate. For a further four trials, the appropriateness of the analysis was judged to be unclear. For the remaining trials, we had substantial reservations regarding the analysis undertaken, which compromise the statistical conclusion validity of the study. These concerns are elaborated in the Discussion section below.

## Discussion

In the present study, we undertook a critical appraisal of the 18 RCTs included in Section 1 of the CultureForHealth report (1), which the authors of the report had not carried out. We analysed the basic characteristics and assessed risks of bias, using the JBI appraisal tool (34). The RCTs were characterised by substantial heterogeneity and high risks of bias, affecting both internal and external validity, and, hence, compromising the claims and recommendations stated within the CultureForHealth report.

### Characteristics of the included RCT

There are key challenges with the basic conduct and reporting of many of the trials included in the CultureForHealth report. Firstly, approximately half of the trials had not been pre-registered, some did not conform to CONSORT guidance, and one trial failed to report ethical approval. These considerations alone should have resulted in extreme caution in even including these trials for consideration in a review. Secondly, the RCTs are characterised by high study heterogeneity regarding all aspects, as highlighted in Table 2. Thirdly, given that the outcomes assessed are mostly based on patient-reported outcomes, the clinical relevance of findings reported is not always clear, with no reference to established cut-off values or MCID. Additionally, distinguishing between primary and secondary outcomes is often not clear. Fourthly, many trials are extremely small and under-powered, and, furthermore, involve predominantly females, so the extent to which the findings could be generalised even to males is questionable. Finally, descriptions of the arts-based interventions used are frequently lacking, including any disease-specific adaptions of the intervention, besides a frequent absence of appropriate and validated checklists for reporting within research on healthcare interventions [e.g., CONSORT (53)].

These challenges severely limit both study replication to confirm or disconfirm findings and the reliable translation of a particular intervention to clinical and/or public health settings. Notably, nine trials are explicitly described as being a “pilot”, “pioneer”, or “exploratory”. As such, our analysis demonstrates that before even considering the assessments of the trials using the JBI tool, there are aspects of the trials which immediately raise questions over external validity and generalisability.

### JBI appraisals of the RCTs

There are several issues affecting all or most of the trials which potentially introduce substantial risks of bias with respect to the outcomes. In all cases, both participants in interventions and those facilitating them were not blinded. This is unavoidable for arts-based activities of all kinds, but nevertheless, awareness introduces the potential for expectation and social desirability biases (54, 55). A further source of non-blinding arises with outcome assessment. Where objective measures are taken by a member of the research team, all trial reports properly describe the assessors as blinded, in all trials but one (52). Some of the outcome measures are participant-reported, which is exclusively the case in six trials (35, 37, 40, 43, 49, 51). Participants were, thus, aware not only of the nature of the intervention, but were also asked to report on the impact of the activity.

A further potential source of bias arises in 15 out of 18 trials as there is no reporting of reliability of data gathered to evaluate the interventions. The JBI appraisal is very clear that the issue is not whether the measures employed had been previously validated, but whether reliability estimates for the data itself are reported. This could readily have been done in two ways: estimates of internal consistency for scales used, or examining correlations between baseline and follow-up assessments, but in only three trials is data reliability reported (35, 44, 50, 52).

In addition to “internal validity”, the JBI appraisal involves assessing “statistical conclusion validity”. For most trials, follow-up is complete (Q10), or levels of attrition are very low, but for four cases, attrition is quite substantial (35, 36, 46, 48). This represents a potential source of bias for these trials, as the sample followed up differs from the initially randomised sample. For four trials, however, an intention-to-treat analysis was explicitly employed, and so participants were analysed as randomised (Q11) (35, 41, 42, 45). For the remainder of the trials where follow up was complete, we judged that participants were analysed as allocated.

The picture is much more varied for Q12, however, on whether the statistical analysis reported was appropriate. Six out of 18 trials were considered to meet exacting demands for statistical analysis (35, 38, 39, 42, 43, 45). Ganzoni et al. (39), for example, are meticulous regarding their account of the intention-to-treat analysis undertaken with reference to a prospective power calculation and use of two-tailed tests. In addition, they include a clear statement of testing for normality and matching the statistical tests employed to the measurement characteristics of the outcome variables.

For the remaining twelve trials, however, there is no prospective power calculation, and in most cases no reference to MCID scores or effect sizes. Accounts of the statistical analysis adopted may appear satisfactory, but with details lacking (e.g., no information on whether *t*-tests were one-tailed or two-tailed), and problems with the reporting of results (e.g., a failure to report *t*-values but only *p*-values).

In two UK trial reports (37, 41), there are also concerns over the details of the statistical strategy adopted, and we discuss the approach adopted in detail to illustrate the threats to validity of the conclusions drawn. Fancourt and Perkins (37), for example, report no differences in depression across three arms of their trial (singing, play, and usual care) after 10 weeks for their total sample of mothers with scores on the Edinburgh Postnatal Depression Scale of ten or greater. They then focus on a smaller sample of mothers with scores of 13 or greater, and, again, find no differences between the trial arms at 10 weeks. Finally, they focus on changes over the first 6 weeks of the trial and find an apparent faster reduction in depression scores over the first 6 weeks of the trial. However, across the first 6 weeks, there was no difference between change for the singing and play groups. Nevertheless, the conclusion reached focuses on the rate of change in the singing group, and it is claimed that “evidence that singing interventions could speed the rate of recovery in women affected by symptoms of PND… could have clinical relevance”. (p. 120) (37).

In the case of Philip et al. (41), the appropriate use of non-parametric techniques where data was not normally distributed, and the intention-to-treat approach are both excellent features of the analysis undertaken, but the use of one-tailed criterion may be criticised as too liberal, as for an exploratory study a two-tailed approach would be recommended (56). This is especially the case given that the reported *p*-value for changes in a measure of depression is 0.049—at the very limit for rejecting the null hypothesis, and, thus, far from convincing.

### Context of the CultureForHealth report

Societies and healthcare systems all over the world face unprecedented challenges in public health, and pressures on health care services and escalating costs (57). Evidence-based medicine, despite extraordinary advances, is also limited in what it can offer people with enduring and progressive health conditions. It is undeniable that cross-sector collaboration and inter-disciplinary working is needed to meet these demands. Improvements in public health will only come about by addressing the root causes of ill-health and health inequities, which has been clear since the seminal work of 19^th^ century social reformers (58), and which currently are amply reinforced by the work of Marmot on the social determinants of health (59).

The arts may be one possible sector to integrate into a holistic strategy for improving health and wellbeing in all sectors of society and across the lifespan (3). The field of arts and health practice and research has indeed made considerable efforts over the last quarter century, and the trials we are considering demonstrate the commitment, passion, and collaborative energy of funding bodies, healthcare professionals, creative artists, and researchers to explore new frontiers in arts and health interventions.

Most of the trials cited in the CultureForHealth report, however, only focus on group singing and dancing, and address specific health issues related to ageing, chronic health conditions, and mental health. As such, the studies may have relevance to addressing just three of the public health challenges identified in the report: the need for greater focus on health promotion, a growing mental health crisis, and an ageing population. In contrast, however, they have little or no relevance to remaining challenges which the report suggests that the arts can help address, such as the need to address the health and wellbeing of young people, addressing health inequalities, and the needs of forcibly displaced people. Moreover, the trials are by no means representative of the international work in arts and health, and many other trials are missing in the report due to its limited search strategy (for example, the report includes three trials concerned with COPD, but misses other key trials published within their time envelope) (60–62). Nevertheless, it is clear that this, albeit selective, corpus demonstrates how global the interest in “creative health” is, with studies as far afield from east to west as China and Brazil, and also in Europe from north to south in the UK and Greece, although no considerations are given in the report on any potential challenges related to drawing conclusions across heterogeneous cultural contexts.

Although scoping reviews do not necessarily involve critical appraisal of the studies included (19, 20), the CultureForHealth report's limited research strategy results in even higher risk of bias even at a basic methodological level. Specifically, the report does not offer critical considerations regarding methodological study type and quality, risks of bias, or descriptions of interventions employed. Moreover, the report does not offer critical considerations on the clinical relevance of outcomes and findings to assess the validity of the evidence claims stated, for example regarding the claim that singing improves respiratory function which, however, has not been demonstrated (62–64).

Moreover, no consideration is given to the dangers of drawing conclusions based on underpowered trials (65). Instead, the authors take findings at face value, and, given the lack of any basic scrutiny, the reporting only meets the very first step in Bloom's taxonomy (66). Moreover, as demonstrated in the present paper, the included primary RCTs do not meet current standards for good practice regarding developing, investigating, reviewing, and evaluating the benefits of healthcare interventions, nor for rigorous synthesis [e.g., the GRADE framework (67, 68)].

Notably, in all 18 trial reports, the authors themselves give due attention to the limitations of their studies and the need for further research. This is, however, not clearly transmitted in the report. Moreover, despite previous criticism regarding the methodology and conclusions of the WHO report (22–24), the CultureForHealth report does not address these concerns and presents no clear aim to enhance the methodological quality of research in the field. Immediate confidence in the criticality of reported findings is further compromised e.g., by the identification of a source described as a “systematic review” on singing and health (1, 69), which, however, is no more than a spreadsheet of selected studies, even depicting incorrect information, e.g., regarding singing and respiratory function (62, 69).

Taken together, the CultureForHealth report (1), similarly to the WHO report (13), does not express any hesitations or cautions regarding conclusions and recommendations stated, nor does it sufficiently consider the following aspects:

- Quality and certainty of the evidence (29, 70);
- Standard frameworks for synthesising the body of evidence regarding complex and health-care interventions, e.g., c.f., the GRADE framework (30, 31, 71);
- Patient safety (32, 33);
- Standards for development/definition of a core outcome set (COS) in health care interventions (72, 73);
- Standards for evaluating the implementation of healthcare interventions, including acceptability, fidelity, feasibility, scalability, and sustainability (74).

Thus, based on findings presented in the present paper and the previous concerns raised, we stress the limitations of scoping reviews and grey literature reports which further perpetuate a lack of scientific rigour and trustworthiness within the field. What is needed are systematic reviews which have undergone thorough external peer-review and which properly assess factors relevant to practice and policy development (22, 75). We welcome the new Lancet Global Series initiative (14–16) and encourage further high-quality, rigorous research, alongside a fruitful and ambitious academic discussion to form a qualified basis for informing policy and practice. However, we have also found a need to express concerns to the initial opinion piece coming from the Jameel Arts & Health Lab (15, 76).

### Strengths and limitations of this study

The present paper builds upon previous critiques of reports and research within arts and health, but is the first study to present in-depth scrutiny of the primary studies on which evidence-claims, conclusions, and recommendations are made in the CultureForHealth report. We have approached the exercise of critique with a proper sense of humility and propriety based on an acknowledged framework, and our paper exemplifies the steps and thoroughness needed for assessment and evaluation of primary studies as a basis for drawing conclusions ahead of formulating any evidence-based recommendations for policy or practice. We did not assess or evaluate other study types included in the report (e.g., quasi-experimental and qualitative studies) and further critical scrutiny may be warranted (23). Given the focus in this paper on the treatment of RCTs, our critique provides a constructive outline and perspectives for future research and provides useful information for readers of the CultureForHealth report.

## Conclusion

The CultureForHealth report substantially fails to meet current standards for good practice regarding evaluation of healthcare interventions. The report is not a suitable foundation for policy or practice recommendations nor for current scaled-up implementation of arts for health initiatives. Future trials should adhere to established high-quality standards for the development and evaluation of healthcare interventions, and robust, critical systematic reviews and meta-analyses are needed as a basis for evaluation of the field before considering policy formulation and practice guidelines.

## Author contributions

MK: Conceptualisation, Data curation, Formal analysis, Investigation, Methodology, Project administration, Resources, Software, Supervision, Validation, Visualisation, Writing – original draft. KG-H: Conceptualisation, Data curation, Formal analysis, Investigation, Methodology, Supervision, Validation, Writing – original draft. CD: Conceptualisation, Methodology, Resources, Validation, Writing – review & editing. GM: Conceptualisation, Methodology, Resources, Validation, Writing – review & editing. JS: Data curation, Resources, Validation, Writing – review & editing. JM: Conceptualisation, Data curation, Formal analysis, Methodology, Resources, Validation, Writing – review & editing. SC: Conceptualisation, Data curation, Formal analysis, Investigation, Methodology, Project administration, Resources, Software, Supervision, Validation, Visualisation, Writing – original draft.
