# Supplementary material for: Is it premature to formulate recommendations for policy and practice, based on culture and health research? A robust critique of the CultureForHealth (2022) report
Source: Front Public Health. 2024 Jul 11;12:1414070. doi: 10.3389/fpubh.2024.1414070 (PMC11287899; doi:10.3389/fpubh.2024.1414070)
Supplement: Supplementary file 1 [file Data_Sheet_1.PDF]

Supplementary material for manuscript:

Is it premature to formulate recommendations for policy and practice, based on culture and health research? A robust critique of the CultureForHealth (2022) report

Supplementary Table 1: A detailed account of JBI coding with illustrative quotations from trial reports

JBI question	Illustrative quotations supporting coding decisions (Rating given before each quotation or in the text)
**Q1/Q2: Randomisation and concealment**	Four trials provide excellent accounts of randomization with concealment which inspire confidence that true randomization was achieved (Feng et al., 2020; Ganzoni et al., 2020; Philip et al., 2020; Cruz-Ferriera, et al., 2015). In each case randomization was undertaken independently of the research team with a specified procedure:<br><br>YES - The randomization list was prepared by an independent statistician from the Singapore Clinical Research Institute (SCRI) using permuted block randomization and a 1:1 allocation ratio. The block length was not made known to the clinical investigators or site personnel. Opaque, sequentially numbered and sealed envelopes were prepared by the independent statistician. Trial coordinator and trial Principal Investigator (LF) opened the randomization envelopes in sequential order to determine treatment allocation. (Feng et al. 2020, p.24807)<br><br>YES - Participants were randomised in a 1:1 ratio to the intervention or standard therapy group using electronically concealed randomisation lists. The randomisation was carried out block-wise by the Clinical Trial Unit of the Department of Clinical Research in Basel. (Ganzoni, et al. 2020, p.2)<br><br>YES - Participants were randomised (1:1) using computer-generated block randomisation lists (Sealed Envelope) block size 4, stratified by Medical Research Council (MRC) breathlessness grade and previous participation in pulmonary rehabilitation. (Philip et al., 2020, p.3)<br><br>YES - Randomization (random table method) was performed by an independent individual from the local health center. The allocation was concealed. (Cruz-Ferriera, et al., 2015, p.840)<br><br>A further three trials gave satisfactory accounts of randomization being conducted independently according to a systematic procedure, but without explicitly noting concealment (Coulton et al., 2015; Lazarou et al., 2017; Marquez et al., 2017, and Huang et al., 2016).<br><br>The following passages, for example, suggest that true randomization took place, with a reasonable assumption of concealment:

	YES - Randomisation was conducted by a secure remote randomisation service independent of the research team. Randomisation employed random permuted blocks of variable length and was stratified by centre and gender. (Coulton, et al., 2015, p.2)<br><br>YES -A stratified block randomisation was performed before the treatment. Age, gender and baseline pain/anxiety characteristics (reflected by the CPT, EEG signals and questionnaire scores) were matched after participants were recruited, and those with outlier scores were excluded. The strict screening process resulted in a relatively small sample size; however, baseline homogeneity was guaranteed. One individual in each block was randomly assigned to the BWM group, the CBT group or the control group, via a computer-generated sequence performed by a Chinese Clinical Trial Registry statistician (Huang, et al., 2016, p.767).<br><br>For the remaining nine trials, the account of the randomisation process, is less satisfactory, with no assurance of concealment The following extracts, for example provide an account of the randomisation procedure but fail to specify who undertook the randomisation:<br><br>UNCLEAR - [Participants] were randomised using SPSS with a 1:1:1 allocation using random block sizes of six, stratified by age of their child and the severity of their EPDS score (Fancourt and Perkins, 2018, p.119).<br><br>UNCLEAR - Patients were randomly allocated to the intervention or control group using a random number table (Liu, et al., 2019, p.726)<br><br>UNCLEAR - Older Latinos who qualified for the study and completed baseline testing were randomly assigned to the dance treatment or to the health education control group using randomization in the Study360™ software (Almedtrac, Inc., Pittsburgh, PA). (Marquez, et al., 2017, p.6).<br><br>Less satisfactory still, are cases where a member of the research team undertook the randomisation of participants:<br><br>UNCLEAR - All participants received detailed information for study participation from a member of the study team on the maternity unit and gave informed written consent. Following that, they filled in the first questionnaire and the study team member allocated them to the control or intervention group via a computer-assisted permuted block randomisation (1:1 allocation ratio). Therefore, the study was not blinded as the participants as well as the team members were aware of the group allocation. (Wulff et al., 2021, p.4).<br><br>UNCLEAR - Participants were assigned to the Tango or Control group by the principal investigator using an online random number generator (Duncan and Earhart, 2012, p.133).<br><br>UNCLEAR - The randomization process was performed by the main researcher. (Foruezandeah, et al., 2020, p.3).<br><br>In the least satisfactory accounts, trial reports state that participants were randomised, without specifying who undertook randomisation or the procedure used:

	UNCLEAR - A total of 120 patients with AS admitted to the People's Hospital of Anhui Province between March 2018 and March 2019 were randomly divided into two groups. The control group was further divided into a routine treatment group and a painting group (Qin, 2020, p.404).<br><br>UNCLEAR – The children were randomly assigned to one of two groups: the music group, children in this group received live music intervention before and during blood sampling, and control group, children in this group received standard medical care (Caprilli, et al., 2007, p.2).<br><br>For Q1, all trials were rated YES or UNCLEAR, as no reports included details which indicated that 'true' randomisation had not been achieved.<br><br>For Q2, however, two trials were given a NO rating (Fancourt and Perkins, 2018; Wulff et al., 2021), as the authors acknowledge lack of concealment:<br><br>NO - Regarding limitations, participants and researchers were not masked to the groups they were allocated to. (Fancourt and Perkins, 2018, p.120)<br><br>NO - Therefore, the study was not blinded as the participants as well as the team members were aware of the group allocation. (Wulff et al., p.4)
**Q3: Equivalence of trial groups at baseline**	Question three in the JBI tool for appraising RCTs asks: 'Were treatment groups similar at the baseline.' This is to be expected given true randomization, although it is not guaranteed, and differences may be apparent for some variables and may even be statistically significant under conditions of random allocation. Also, if sample sizes are small apparent differences may not be significant for this reason. Barker et al. (2023) identifies differences at baseline as a potential source of bias, and caution that reviewers should 'not only consider the P value for the statistical testing of differences' at baseline.' (p.499)<br><br>One trial under consideration does not report baseline data (Caprilli et al., 2007), as the study involves the randomization of children due to undergo venipuncture in a hospital setting, and only outcome assessment of observed anxiety levels is reported.<br><br>For 13 trials (YES), authors report that baseline demographic details and outcome variables do not differ significantly between or across trial arms, and this was judged to be convincing (Coulton et al., 2015; Fancourt and Perkins, 2018; Feng et al., 2020; Liu et al., 2019; Pongan et al., 2017; Wulff et al., 2021; Cruz-Ferreria et al., 2015; Duncan and Earhart, 2012; Kalsatou et al., 2015; Huang et al., 2016; Qin, 2020; Foruezandeah et al., 2020).<br><br>For two trials, however, groups noticeably differ on some variables even though the differences are not statistically significant. The Bonilha et al. (2009) trial, for example, involves only 15 participants in each arm, and they acknowledge that the mean quality of life measure was higher in the control group than the singing group:

	UNCLEAR - The groups did not differ significantly regarding their basal clinical or functional features (Table 1). Although the mean QoL score of the Control Group was higher than that of the Singing Group, the difference did not reach statistical significance (p = 0.06). (Bonilha et al., 2009, p.3)<br><br>Similarly, the Philip et al. (2020) trial (UNCLEAR) involves only nine participants in each arm, and while groups do not differ statistically at baseline on the measures compared, there are marked differences between the groups in sex composition (33% females in the singing group and 66% in the usual care group) some SF-36 sub-scales (e.g. SF-36 Role limitation, physical – mean of 50 for the singing group vs mean of 25 for the usual care group), and scores on the GAD-7 measure of anxiety (median of 2 in the singing group and median of 5 in the usual care group).<br><br>Less reassuring regarding equivalence of groups at baseline are accounts of three trials in which statistical comparisons are not reported (e.g. Ganzoni et al., 2020; Marquez et al., 2017) or reporting is incomplete (e.g. Lazarou et al., 2017). (ALL UNCLEAR)
**Q4/Q5: Blinding of participants and personnel delivering interventions**	Question 4 in the JBI scale asks: 'Were participants blind to treatment assignment?' A central feature of medical trials is that participants are blind to the interventions received. This is critical as Barker et al. (2023) note that participants 'who are aware of their allocation to either the treatment or control may behave, respond, or react differently to their assigned treatment…' (p.499) This potentially introduces a substantial risk of bias if blinding does not occur or is compromised. For all the trails considered here, however, blinding is not possible. Participants engaged in singing, dancing or musical activities will clearly be aware of the nature of the intervention and are very likely to entertain the assumption that the activity is offered as a form of treatment or therapy for challenges they are experiencing to their health or wellbeing. The same issues arise with respect to personnel delivering interventions. Question 5 in the JBI tool asks: 'Were those delivering the treatment blind to treatment assignment?' (p.499) This is important in medical trials because personnel delivering a treatment vs control, or two alternative treatments, could behave differentially thus introducing a potential source of bias. 'Blinding of those delivering treatment' as Barker et al. (2023) note, 'is used to minimize this risk.' (p.499) However, a musician facilitating a singing group, or a dance artist leading dance activity, cannot but be aware of their role in delivering an intervention.<br><br>Accordingly, Table 2 [JBI ratings] shows that lack of blinding of participants and those delivering interventions is a potential source of bias in all the trials considered (NO). Research teams will, of course, be fully aware of this but in only four trial reports is the lack of participant blinding acknowledged (Fancourt and Perkins, 2018; Ganzoni et al., 2020; Philip et al', 2020; Wulff et al., 2021).<br><br>Ganzoni et al. (2020) are clear that lack of blinding compromises the interpretation of their patient reported outcome measure: '… given that our study was not blinded, the interpretation of the quality of life is limited.' Whereas Fancourt and Perkins (2018) appear to discount the possible bias involved in lack of blinding on the grounds that participants were not informed of the study hypothesis:<br><br>NO - Regarding limitations, participants and researchers were not masked to the groups they were allocated to. However, women were not informed that the study hypothesis involved singing having significantly different results to the play group, so having significantly different results to the play group, so the fact that there were no improvements in the play group compared with the control would seem to suggest results were not entirely driven by placebo (Fancourt and Perkins, 2018, p.120).

**Q6: Identical treatment of groups apart from the intervention(s) tested**	Question 6 of the JBI assessment tool asks: Were treatment groups treated identically other than the intervention of interest. Table 2 [JBI ratings] indicates that our judgements were that this condition was met in all the trials considered (IN ALL CASES YES). We could find no indication in any of the trial reports to suggest that there were 'other exposures or treatments occurring at the same time as (…) the treatment or intervention of interest' (Barker et al., 2023, p.499).
**Q7: Blinding of Outcome Assessors**	Question 7 of the JBI tool asks: Were outcome assessors blind to treatment assignment?' Barker et al. (2023) links this question with the blinding of assessors and the blinding of participants and those delivering interventions, as in all cases awareness can result in potential bias: 'If those assessing the outcomes are aware of participant allocation to either treatment or control, they may treat participants differently compared with those who remain unaware…' (p.500). In most of the trials under consideration, a battery of outcome measures is employed, some of which are objective physiological and behavioural measures, and others psychological assessment (e.g., anxiety, depression, quality of life). A crucial factor to consider here is that patient reported outcome measures through completion of questionnaires cannot be blind, nor can subjective assessments gathered by structured interviews (where the interviewer may be blind). For this reason, most of the judgements reported for 'blinding' in Table 1 are mixed – both YES and NO – where objective assessor blinded measures were combined with subjective patient-reported outcomes.For six trials (Coulton et al., 2015; Fancourt and Perkins, 2018; Wulff et al., 2021; Caprilli et al., 2007 and Qin, 2020) all outcomes are patient reported (NO IN ALL CASES)<br><br>Most trial reports explicitly note the blinding of assessors for both objective and subjective outcome measures, even though for patient-reported outcomes, the participants are not blind. The following give examples of such accounts:<br><br>(YES, BUT NO FOR PATIENT REPORTED OUTCOMES) Trained research assistants conducted the neuropsychological assessment to collect cognitive data at baseline, 12 months, and 24 months. Training to all research staffs was provided by a single instructor (LF) to ensure consistency and data quality. (…) Outcome assessors and laboratory staff were blinded to allocation status of trial participants. (Feng et al., 2020, p.24805).<br><br>(YES, BUT NO FOR PATIENT REPORTED OUTCOMES) The primary endpoint was the difference in change in maximum inspiratory pressure (ΔMIP%predicted). (…) MIP and MEP were measured by experienced respiratory technicians at the Department of Pneumology, University Hospital Basel. The technicians were blinded to the randomisation. (Ganzoni et al., 2020, p.3).<br><br>(YES, BUT NO FOR PATIENT REPORTED OUTCOMES) Chronic pain, anxiety, depression, and quality of life were assessed before, after, and 1 month after the sessions. Cognitive abilities were assessed before and after interventions. (…) All principal outcome measures were assessed by an independent neuropsychologist in each center, who was different from the practitioner conducting SI or PI. (…) Both the neuropsychologist and the research team members were blind to the intervention type. (Pongan et al., 2017, p.667)<br><br>(YES, BUT NO FOR PATIENT REPORTED OUTCOMES) Physical fitness (strength, aerobic endurance, flexibility, motor agility/dynamic balance, and body composition) and life satisfaction were evaluated at the local health center at baseline, 12 weeks, and 24 weeks by an assessor who was blinded to group allocation. (Cruz-Ferreria et al., 20115, p.840).

	(YES, BUT NO FOR PATIENT REPORTED OUTCOMES) The primary outcome measure was the Movement Disorders Society–Unified Parkinson Disease Rating Scale 3 (MDS-UPDRS-3). (…) Secondary outcome measures were the MDS-UPDRS-1, MDS-UPDRS-2, MiniBESTest balance test; Freezing of Gait questionnaire (FOG_Q); 6-Minute Walk Test (6MWT); gait velocity for comfortable forward, fast as possible forward, dual task, and backward walking; and Nine-Hole Peg Test (9HPT). (…) For each assessment (…) participants were assessed by the same rater, a physical therapist (RPD), who was blinded to group. (Duncan and Earhart, 2012, p.133)<br><br>Only Lazarou et al. (2017), however, notes that participants were explicitly asked not to reveal their group allocation to assessors at post-test:<br><br>(YES, BUT NO FOR PATIENT REPORTED OUTCOMES) Due to the nature of the therapy programs, blinding of the participants and instructor is not possible. However, all the independent evaluators are blinded with respect to group allocation, and the participants are not informed of primary outcome measure or the study hypothesis. To maintain group allocation confidential, participants are requested prior to each assessment phase to not reveal allocation or therapy content to the evaluators. Participants scheduled for qualitative studies are told that they must not talk to the evaluators about participation in interviews and neuropsychological assessment. Furthermore, the interviews and focus groups were performed in a way that does not reveal participants' allocation. (Lazarou et al., 2017, p.492)<br><br>Only three trial reports explicitly note a lack of blinding of researchers and assessors, that could potentially bias outcome assessments (Fancourt and Perkins, 2018; Wulff et al., 2021; Caprilli et al., 2007). Caprilli et al. (2007), for example states:<br><br>NO - … the observers [using an observation scale of behavioural distress] would be aware of the assignment because they would see/hear the musicians or recognize their absence. Therefore, this could have influenced their observations. The effect of such a bias would be to lower the mean distress and pain in the music condition (…). We acknowledge this limitation, and it would be desirable to obtain some blind outcome measures, if possible, in future studies. (Caprilli et al., 2007, p.4).
**Q8: Equivalence of measured outcomes for treatment groups**	Question eight in the JBI tool asks: Were outcomes measured in the same way for treatment groups?' In all cases, our assessment was YES.
**Q9: Reliability of outcome assessments**	Question 9 in the JBI assessment tool asks: 'Were outcomes measured in a reliable way?' The guidance for this question states:<br><br>'Reviewers should check the details about the reliability of the measurement used, such as the number of raters, the training of raters, and the reliability of the intra-rater and inter-raters with the study (not as reported in external sources). This question is about the reliability of the measurement performed in the study, and not about the validity of the measurement instrument/scales used in the study.' (Barker, et al., 2023, p.500)

	As such, the guidance is stringent and demanding, and only three out of the 18 trials reviewed provide any evidence on the reliability of some, but not all of the data reported (Cruz-Ferreria, et al., 2015; Caprilli, et al., 2007; Foruezandeah et al., 2020). Cruz-Ferreria, et al., (2015), for example, claim that both the physical assessment test, and the life satisfaction scale employed have previously been shown to be reliable and valid, but it is only for life satisfaction of a reliability estimate for the sample investigated is reported:<br><br>> YES / NO - The physical fitness parameters were evaluated using the Senior Fitness Test (Rikli & Jones, 1999). This test has been shown to be a reliable and valid measure of physical fitness (Rikli & Jones, 1999), and it comprises the major physical fitness parameters required for independent daily living. (…) The Satisfaction with Life scale (Diener, Emmons, Larsen, & Griffin, 1985) was used to measure life satisfaction. This scale is a valid and reliable measure of life satisfaction (Pavot, Diener, Colvin, & Sandvik, 1991). The scores ranged from 5 to 35, and an increase in the score corresponded with a positive improvement. For the present sample, the internal consistency, as evaluated by Cronbach's a reliability coefficient, was .769. (Cruz-Ferreria, et al., 2015, p.844)<br><br>In Foruezandeah et al. (2020), information is provided on the reliability of the observational assessments made of children's pre-operative anxiety:<br><br>> YES - The children's anxiety was measured before and 5 min after the intervention by a person who was blinded about the intervention by using the modified Yale Preoperative Anxiety Scale (mYPAS), which consists of 22 items in five domains. (…) The validity and reliability of this scale have been confirmed in different studies [17–19].(…) In the present study, the reliability of the scale was confirmed with a Cronbach's alpha of 0.85. (Forouzandeah, et al., 2020, p.3).<br><br>More commonly, however, authors claim that the outcome measures employed have acceptable levels of reliability and validity, although this is not confirmed for the data gathered during the trial (Coulton, et al., 2015; Liu, et al., 2019; Duncan and Earhart, 2012; Kalsatou, et al., 2015; Marquez, et al., 2017; Qin, 2020).<br><br>Coulton et al. (2015) state the following, regarding the primary outcome measure used in their trial of community singing for older people:<br><br>> NO - The primary outcome measure was mental health-related quality of life assessed by the York SF1219 at 6 months post randomisation. The SF12 contains 12 items addressing both mental and physical health components of quality of life and has established psychometric properties including reliability, validity and sensitivity to change. (Coulton, et al., 2015, p.2).<br><br>Liu et al. (2019) also claim satisfactory psychometric properties for their primary outcome, and go further by indicating that the UK-developed scale has been validated with Chinese patients:<br><br>> NO - Data were collected by means of self-administered questionnaires. The HADS-D subscale was used for assessment of depressive symptoms. This scale was developed by Zigmond et al. [35] and includes seven items. Each item is scored on a four-

	point (0–3) scale; the total score ranges from 0 to 21. A score of 8 or higher is indicative of depression. HADS has been shown to have a high sensitivity and specificity in Chinese patients Cronbach's alpha for HADS-D: 0.79 [36]. (Liu, et al., 2019, p.728)<br><br>Qin (2020) reports on both the reliability and internal consistency of the Generic Quality of Life Inventory used in her study of music therapy with patients affected by a severe form of arthritis, but does not report information for the data gathered:<br><br>NO - Generic Quality of Life Inventory (GQO-LI-74) (Chen et al. 2007) was used in this study. The test–retest reliability of the inventory is 0.84–0.93, and the internal consistency reliability is 0.66–0.69. The inventory has good content validity, construct validity, and criterion validity. (Qin, 2020, p.405)<br><br>The remaining nine trials, however, include no mention of the reliability and validity of the outcome measures they use (Bonhila et al., 2009; Fancourt and Perkins, 2018; Feng et al., 2020; Ganzozi et al., 2020; Philip et al., 2020; Pongan et al., 2017; Wulff, et al., 2021; Lazarou, et al., 2017; Huang, et al., 2016) (NO IN ALL CASES). It should be noted, however, that in most of these trials standardised objective assessment procedures and previously validated scales were employed, and it would be reasonable to assume that they satisfactorily employed.
**Q10: Post randomisation attrition**	Question 10 asks: 'Was follow-up complete and, if not, were differences between groups in terms of their follow up adequately described and analyzed?'<br><br>This item in the JBI tool is essentially concerned with attrition and how this is dealt with. As Barker et al. (2023) state: 'Because RCTs are not perfect, there is almost always post-assignment attrition, and the focus of this question is on appropriate exploration of post-assignment attrition.' As such, this question is linked with Q11 which asks whether an 'intention-to-treat' analysis was undertaken, as this is a key analytical strategy for dealing with attrition in effectiveness studies. Barker et al. (2023) explicitly states that 'Question 10 is *not* about intention-to-treat analysis' (p.501) (emphasis in the original).<br><br>Among the 18 trials considered, eight reported no attrition, and five reported attrition that was very low. Fancourt and Perkins (2018), for example, report an attrition rate of 9% over the full sample and this is the only report that refers to a comparison between participants followed up and those who dropped out: 'There were no differences across any of the baseline variables measured between those who did complete and those who did not complete data collection.' (p.119).<br><br>In the case of two trials (Coulton, et al., 2015; Duncan and Earhart, 2012) (NO IN BOTH CASES), there was substantial dropout over the course of the trial. Coulton et al. state that: 'Follow-up rates at 3 and 6 months were 222 (86%) and 204 (79%), respectively, and no differential follow-up rate between the groups was observed' meaning that attrition over the course of the trial was 21%. No comparisons are made between those who completed the study and those who dropped out, but attrition was dealt with by an intention to treat analysis. Duncan and Earhart state that 'Over the 12-month study, there was a 37% attrition rate in the Control group and a 50% attrition rate within the Tango group.' As with the Coulton et al. study, no comparisons are made between those who dropped out and those who completed the study, but this attrition was dealt with by an intention to treat analysis based on participants who were assessed at the three-month follow-up.

	Two further trials had substantial attrition and analysis is based on those completing the trail. For the Lazarou et al. (2015) (NO) trial attrition in the dance intervention group was 26%, over ten months, but the CONSORT diagram is incomplete, and no comparison is made between those dropping out and those completing. The statistical analysis of baseline data and at follow-up is based on those completing the study. In the case of Marquez et al. (2017) (NO), the total attrition rate was 17% over four months with no comparisons made between those dropping out and those completing the intervention.
**Q11: Participants analysed as allocated (Intention-to-Treat analysis)**	The first ten questions of the JBI tool refer to risk of bias to the internal validity of the trial. Now, with questions 11 to 13 the focus is on 'statistical conclusion validity', that is whether we can be confident that the statistical analysis undertaken involves no potential bias affecting the conclusions drawn.<br><br>Q11 of the JBI tool asks: 'Were participant analyzed in the groups to which they were randomised?' and they explain that the focus here is whether an 'intention to treat' analysis was undertaken. Barker et al. (2023) explains further:<br><br>> The ITT analysis is a type of statistical analysis recommended in the Consolidated Standards of Reporting Trials (CONSORT) statement on best practices in trials reporting, and it is considered a marker of good methodological quality of the analysis of a randomized trial. The ITT is estimating the effect of offering the intervention (ie, the effect of instructing the participants to use or take the intervention); the ITT is not estimating the effect of receiving the intervention of interest. (Barker et al., 2023, p.501).<br><br>Of the 18 trials, six explicitly state that an Intention to Treat analysis was performed (Coulton et al., 2015; Feng et al., 2020; Ganzoni et al., 2020, Liu et al., 2019; Pongan et al., 2017; Duncan and Earhart, 2012) (YES IN ALL CASES). Of these trials, only Pongan et al. (2017) reports both an ITT analysis and a per protocol analysis reflecting engagement with the interventions investigated:<br><br>> YES - The primary data analysis was by intention-to-treat (ITT), carried out using multiple imputation dataset, with full analysis of all patients as randomized and completing the baseline assessment. The analysis was then completed by a 'per protocol' (PP) approach conducted on the patients who completed at least 6 SI or PI sessions, representing the 'treatment maintenance' sample, and reflecting usual clinical practice for AD patients participating to group interventions. (Pongan et al., 2017, pp.667-8).<br><br>For nine trials where little or no attrition occurred (ADD), a YES rating was also given.<br><br>For three trials (Bonilha et al., 2009; Lazarou et al., 2017; Marquez et al.,2017), where an intention-to-treat analysis was not undertaken, and attrition was judged to be substantial, a rating of NO was given.
**Q12: Appropriate statistical analysis**	Question 12 asks: Was appropriate statistical analysis used? Barker et al. (2023) explains the focus of this question in detail:<br><br>> Low statistical power and the violation of assumptions of statistical tests are 2 important threats that weaken the validity of inferences about the statistical relationship between the cause and the effect. Reviewers should check the following aspects: if

	the assumptions of statistical tests were respected; if appropriate statistical power analysis was performed; if appropriate effect sizes were used… (Barker et al., 2023, p.501)<br><br>In assessing the 18 trials under consideration, texts were searched for references to power, effect sizes and discussion of assumptions made regarding the data gathered (e.g., measurement level, normality etc.) In addition, we considered whether trials employed estimates of 'minimal clinically important change' scores for primary outcome measures, referred to the use of two-tailed tests, and reported confidence intervals. Our assessments in Table 2 (JBI ratings) reflect a combination of these criteria.<br><br>Five out of 18 trials were considered to meet exacting demands for statistical analysis (Coulton et al., 2015; Ganzoni, 2020; Pongan et al., 2017; Duncan and Earhart 2012; Caprilli et al., 2007) . The following quotations illustrate the approaches adopted. Coulton et al. (2015), for example, refers to a prospective power calculation; an estimate of the MCID on the primary outcome measure; the use of two-tailed tests, an intention-to-treat analysis, and the use of statistical techniques to control for baseline differences. What is missing, however, is an account of measurement level and data distribution characteristics:<br><br>YES - The primary outcome measure was the mental health component of quality of life measured by the SF12 at 6-months post-randomisation. A clinically important difference on this dimension is estimated as a difference of five points between intervention and control group, equivalent to a medium effect size difference of 0.5. To detect this difference using a two-tailed test, alpha of 0.05 and power at 80% requires 63 participants in each of the two arms, a total of 126 participants. (…) As a study of effectiveness, the primary analysis was by intention-to-treat where participants were analysed as part of their allocated group irrespective of the actual treatment received.<br><br>The primary outcome measure, SF12 mental components at 6 months, was analysed by an analysis of covariance adjusting for baseline age and gender which are known covariates. As the intervention involved groups, we adjusted the analysis using the Huber-White sandwich estimation technique to generate robust standard errors. Secondary outcomes were analysed in a similar manner. (Coulton et al. 2015, pp.2-3).<br><br>Ganzoni et al. (2020) are similarly meticulous their account of the intention-to-treat analysis undertaken with reference to a prospective power calculation and use of two-tailed tests. In addition, they include a clear statement of testing for normality and matching the statistical tests employed to the measurement characteristics of the outcome variables:<br><br>YES - Based on previous data, we estimated that a sample size of 11 individuals in each group would have a power of 80% to detect a 10% difference in MIP % predicted for a two-sided alpha set at 0.05. (…) Data were analysed using SPSSR for Windows (version 23, SPSS, Chicago) and tested for normality with the Kolmogorov-Smirnov test. Descriptive data for continuous variables were presented as means with standard deviations (SDs) or as medians with interquartile ranges (IQRs) as appropriate. For comparison between groups, continuous variables were evaluated using the independent Student t-test and proportions were evaluated using chi-square or Fischer-exact tests as appropriate. (Ganzoni et al., 2020, p.3).

Duncan and Earhart (2012) also report a prospective power calculation based on an estimate of the MCID for their main outcome measure. An intention-to-treat analysis was pursued with careful use of techniques to undertake post-hoc comparisons:

> YES - Power analyses based on data from previous published tango and the minimal clinically important difference (MCID) in the UPDRS-327 indicated a need for approximately 30 subjects per group to have 80% power at *P* = .05. (…) T tests compared baseline demographic and disease severity characteristics between groups (*P* = .05). All other data were analyzed using 2-way repeated-measures ANOVAs with group (Tango or Control) and time (baseline, 3, 6, or 12 months) as factors (*P* = .05). When appropriate, Tukey–Kramer multiple comparison post hoc tests were used to determine specific differences between groups within a given time point and within a group across time points. An intent-to-treat analysis with the last observation carried forward was employed with any participants who completed the baseline visit and at least 1 other evaluation. (Duncan and Earhart, 2012, p.134).

For two trials (Feng, et al., 2020; Wulff et al., 2021) a prospective power calculation was undertaken, but the target sample size was not achieved and therefore the study was under-powered, even though the trials were relatively large. For these trials a rating of UNCLEAR was given.

For the remaining twelve trials, however, there was no prospective power calculation, and in most cases no reference to MCID scores or effect sizes. Accounts of the statistical analysis adopted may appear satisfactory, but with details lacking (e.g. no information on whether t-tests were one-tailed or two-tailed), and problems with the reporting of results (a failure to report t-but only p-values).

In two UK trial reports, there are also concerns over the details of the statistical strategy adopted. Fancourt and Perkins (2018), for example, report no differences in depression across their three arms of trial (singing, play and usual care) after ten weeks for their total sample of mothers with scores on the Edinburgh Postnatal Depression Scale of ten or greater. They then focus on a smaller sample of mothers with scores of thirteen or greater, and again find no differences between the trial arms at ten weeks. So, they then focus on changes over the first six weeks of the trial and find and apparent faster reduction in depression scores over the first six weeks of the trial. At this point comparisons are made using change scores across the groups, and this is how they report the result:

> NO - To explore which group differed across this period of significance, we ran ANOVAs of the change from baseline to week 6, which confirmed the significant difference between groups (F2,72 = 3.93, P = 0.024, η2 = 0.10), with post hoc tests with Bonferroni corrections demonstrating that the singing group had a significantly faster improvement than the control group (mean difference −2.83, s.e. = 1.06, 95% CI −5.44 to −0.22, P = 0.029, d = 0.78) but not the play group (mean difference −2.03, s.e. = 1.05, 95% CI −4.61 to 0.54, P = 0.17, d = 0.56), with no difference between the play and control group (mean difference −0.80, s.e. = 1.13, 95% CI −3.57 to 1.97, P > 0.99, d = 0.20). (Fancourt and Perkins, 2018, p.120).

In other words, across the first six weeks, there was no difference between change for the singing and play groups. Nevertheless, the conclusion reached focuses on the rate of change in the singing group:

	> This study supports findings from previous studies showing that symptoms of PND improve over time. However, mothers involved in the singing group had a significantly faster decrease in their symptoms. (…) However, early remission from PND has been associated with reduced effects on both mother and baby. Consequently, evidence that singing interventions could speed the rate of recovery in women affected by symptoms of PND could have clinical relevance. (Fancourt and Perkins, 2018, p.120).<br><br>The approach to the analysis may be criticised as an example of data trawling, and 'hypothesising after the results are known' or HARKING.<br><br>Philip et al. (2020) adapts a one-tailed test for assessing changes in their small trial of singing (in person and then online) for people with COPD:<br><br>> NO - Differences in outcomes between study arms were evaluated using one-tailed t-tests for superiority of SLH over UC or the Wilcoxon rank-sum (WRS) test where data were not normally distributed. Analyses were carried out using Stata V.14 (StataCorp) on an intention-to-treat basis. (Philip et al., 2020, p.4).<br><br>The appropriate use of non-parametric techniques where the data was not normally distributed, and the intention-to-treat approach are both excellent features of the analysis undertaken, but the use of one-tailed criterion may be criticised as too liberal, when in an exploratory study, a two-tailed approach would be recommended. This is especially the case given that the reported p-value for changes in a measure of depression is 0.049 – at the very limit for rejecting the null hypothesis.
**Q13: Appropriateness of the RCT design**	The final question of the JBI appraisal tool asks: 'Was the trial design appropriate and any deviations from the standard RCT design (…) accounted for in the conduct and analysis of the trial? None of the alternatives to a standard parallel group trial design they mention (crossover trial, cluster trial and stepped-wedge trial) are represented among the 18 trials under consideration.<br><br>Trials do vary in the composition of the trial arms: an intervention group and usual activities control group (Coulton et al., 2015); two intervention groups without a usual treatment control (e.g., Feng et al., 2020), or two intervention groups with a usual treatment control (e.g. Fancourt and Perkins, 2018).<br><br>We therefore give a YES judgement for all the trials in Table 2.

References

Bonilha, A. G., Onofre, F., Vieira, M. L., Prado, M. Y. A., & Martinez, J. A. B. (2009). Effects of singing classes on pulmonary function and quality of life of COPD patients. International Journal of Chronic Obstructive Pulmonary Disease, 4, 1–8. https://www.ncbi.nlm.nih.gov/pmc/articles/PMC2672787/pdf/copd-4-001.pdf

Caprilli, S., Anastasi, F., Grotto, R. P. L., Scollo Abeti, M., & Messeri, A. (2007). Interactive music as a treatment for pain and stress in children during venipuncture: a randomized prospective study. Journal of Developmental and Behavioral Pediatrics, 28(5), 399–403. https://doi.org/10.1097/DBP.0b013e31811ff8a7

Coulton, S., Clift, S., Skingley, A., & Rodriguez, J. (2015). Effectiveness and cost-effectiveness of community singing on mental health-related quality of life of older people: randomised controlled trial. The British Journal of Psychiatry: The Journal of Mental Science, 207(3), 250–255. https://doi.org/10.1192/bjp.bp.113.129908

Cruz-Ferreira, A., Marmeleira, J., Formigo, A., Gomes, D., & Fernandes, J. (2015). Creative dance improves physical fitness and life satisfaction in older women. Research on Aging, 37(8), 837–855. https://doi.org/10.1177/0164027514568103

Duncan, R. P., & Earhart, G. M. (2012). Randomized controlled trial of community-based dancing to modify disease progression in Parkinson disease. Neurorehabilitation and Neural Repair, 26(2), 132–143. https://doi.org/10.1177/1545968311421614

Fancourt, D., & Perkins, R. (2018). Effect of singing interventions on symptoms of postnatal depression: Three-arm randomised controlled trial. British Journal of Psychiatry, 212(2), 119–121. https://doi.org/10.1192/bjp.2017.29

Feng, L., Romero-Garcia, R., Suckling, J., Tan, J., Larbi, A., Cheah, I., Wong, G., Tsakok, M., Lanskey, B., Lim, D., Li, J., Yang, J., Goh, B., Teck, T. G. C., Ho, A., Wang, X., Yu, J.-T., Zhang, C., Tan, C., Chua, M., Li, J., Totman, J., Wong, C., Loh, M., Foo, R., Tan, C.H., Goh, L.G., Mahendran, R., Kennedy, B.K., & Kua, E.-H. (2020). Effects of choral singing versus health education on cognitive decline and aging: a randomized controlled trial. Aging, 12(24), 24798–24816. https://doi.org/10.18632/aging.202374

Forouzandeh, N., Drees, F., Forouzandeh, M., & Darakhshandeh, S. (2020). The effect of interactive games compared to painting on preoperative anxiety in Iranian children: A randomized clinical trial. Complementary Therapies in Clinical Practice, 40, 101211. https://doi.org/10.1016/j.ctcp.2020.101211

Ganzoni, C., Arslani, K., Pfister, O., Freese, M., Strobel, W., Mueller, C., & Tobler, D. (2020). Choir singing improves respiratory muscle strength and quality of life in patients with structural heart disease – HeartChoir: a randomised clinical trial. Swiss Medical Weekly, 150, w20346. https://doi.org/10.4414/smw.2020.20346

Huang, R., Wang, J., Wu, D., Long, H., Yang, X., Liu, H., Gao, X., Zhao, R., & Lai, W. (2016). The effects of customised brainwave music on orofacial pain induced by orthodontic tooth movement. Oral Diseases, 22(8), 766–774. https://doi.org/10.1111/odi.12542

Kaltsatou, A., Kouidi, E., Fountoulakis, K., Sipka, C., Theochari, V., Kandylis, D., & Deligiannis, A. (2015). Effects of exercise training with traditional dancing on functional capacity and quality of life in patients with schizophrenia: a randomized controlled study. Clinical Rehabilitation, 29[1] A. K(9), 882–891. https://doi.org/10.1177/0269215514564085

Lazarou, I., Parastatidis, T., Tsolaki, A., Gkioka, M., Karakostas, A., Douka, S., & Tsolaki, M. (2017). International ballroom dancing against neurodegeneration: A randomized controlled trial in Greek community-dwelling elders with mild cognitive impairment. American Journal of Alzheimer's Disease and Other Dementias, 32(8), 489–499. https://doi.org/10.1177/1533317517725813

Liu, H., Song, M., Zhai, Z.-H., Shi, R.-J., & Zhou, X.-L. (2019). Group singing improves depression and life quality in patients with stable COPD: a randomized community-based trial in China. Quality of Life Research: An International Journal of Quality of Life Aspects of Treatment, Care and Rehabilitation, 28(3), 725–735. https://doi.org/10.1007/s11136-018-2063-5

Marquez, D. X., Wilson, R., Aguiñaga, S., Vásquez, P., Fogg, L., Yang, Z., Wilbur, J., Hughes, S., & Spanbauer, C. (2017). Regular Latin dancing and health education may improve cognition of late middle-aged and older Latinos. Journal of Aging and Physical Activity, 25(3), 482–489. https://doi.org/10.1123/japa.2016-0049

Philip, K. E., Lewis, A., Jeffery, E., Buttery, S., Cave, P., Cristiano, D., Lound, A., Taylor, K., Man, W. D.-C., Fancourt, D., Polkey, M. I., & Hopkinson, N. S. (2020). Moving singing for lung health online in response to COVID-19: experience from a randomised controlled trial. BMJ Open Respiratory Research, 7(1). https://doi.org/10.1136/bmjresp-2020-000737

Pongan, E., Tillmann, B., Leveque, Y., Trombert, B., Getenet, J. C., Auguste, N., Dauphinot, V., El Haouari, H., Navez, M., Dorey, J.-M., Krolak-Salmon, P., Laurent, B., & Rouch, I. (2017). Can musical [singing] or painting interventions improve chronic pain, mood, quality of life, and cognition in patients with mild Alzheimer's Disease? Evidence from a randomized controlled trial. Journal of Alzheimer's Disease: JAD, 60(2), 663–677. https://doi.org/10.3233/JAD-170410

Qin, Y. (2020). Effect of music therapy Intervention on physical functions and mental health of patients with Ankylosing Spondylitis. Psychiatria Danubina, 32(3–4), 403–410. https://doi.org/10.24869/psyd.2020.403

Wulff, V., Hepp, P., Wolf, O. T., Fehm, T., & Schaal, N. K. (2021a). The influence of maternal singing on well-being, postpartum depression and bonding – a randomised, controlled trial. BMC Pregnancy and Childbirth, 21(1), 501. https://doi.org/10.1186/s12884-021-03933-z
