# Supplementary material for: Is it premature to formulate recommendations for policy and practice, based on culture and health research? A robust critique of the CultureForHealth (2022) report
Source: Front Public Health. 2024 Jul 11;12:1414070. doi: 10.3389/fpubh.2024.1414070 (PMC11287899; doi:10.3389/fpubh.2024.1414070)
Supplement: Supplementary file 2 [file Data_Sheet_2.pdf]

Supplementary material for manuscript:

Is it premature to formulate recommendations for policy and practice, based on culture and health research? A robust critique of the CultureForHealth (2022) report

List of useful websites where readers can find further information on the subject

Weblinks to reports and guidelines

Culture Action Europe. CultureForHealth [Internet]. 2021 [cited 2024 Mar 3]. Available from: https://cultureactioneurope.org/projects/cultureforhealth/

Zbranca, R., Dâmaso, M., Blaga, O., Kiss, K., Dascșl, M. D., Yakobson, D., & Pop, O. CultureForHealth Report. Culture's contribution to health and well-being. A report on evidence and policy recommendations for Europe. CultureForHealth. Culture Action Europe. 2022. ISBN: 978-2-9603183-0-2 [Internet]. Available from: https://www.cultureforhealth.eu/app/uploads/2023/02/Final_C4H_FullReport_small.pdf

Dâmaso, M., Dowden, S., Smith, C. Culture for Health and Well-being Compendium - A Guide for Practitioners [Internet]. CultureForHealth. Culture Action Europe; 2023 Jun. Available from: https://www.cultureforhealth.eu/app/uploads/2023/06/C4H_Compendium_V4_LP.pdf

Fancourt D, Finn S. What is the evidence on the role of the arts in improving health and well-being? A scoping review. [Internet]. WHO Regional Office for Europe; Copenhagen; 2019. (Health Evidence Network (HEN) synthesis report No. 67.). Available from: https://www.who.int/europe/publications/i/item/9789289054553

The Jameel Arts & Health Lab. Jameel Arts & Health Lab - Measurably improving health and wellbeing through the arts [Internet]. Available from: https://www.jameelartshealthlab.org

Gordon-Nesbitt, R. Older and wiser? Creative ageing in the UK 2010-19 [Internet]. King's College London; 2019. Available from: https://baringfoundation.org.uk/resource/older-and-wiser-creative-ageing-in-the-uk-2010-19/

Dowlen, R. Vision paper: Culture, health and wellbeing [Internet]. Centre for Cultural Value; 2023. Available from: https://www.culturehive.co.uk/CVIresources/culture-health-and-wellbeing/

Weblinks to collaborating centres

Social Biobehavioural Research Group, University College London. WHO Collaborating Centre for Arts and Health [Internet]. Available from: https://sbbresearch.org/projects/who-collaborating-centre-for-arts-and-health/

Edge Hill University. Research Centre for Arts and Wellbeing [Internet]. Available from: https://sites.edgehill.ac.uk/rcaw/about-the-centre/

The Jameel Arts & Health Lab. Jameel Arts & Health Lab - Measurably improving health and wellbeing through the arts [Internet]. Available from: https://www.jameelartshealthlab.org

Institute of Health Equity. Available from: https://www.instituteofhealthequity.org/home

Weblinks for launch events and meetings

WHO expert meeting on prevention and control of noncommunicable diseases: learning from the arts. [Internet]. 2022 Dec 15; Opera House Budapest, Hungary. Available from: meeting report: https://iris.who.int/handle/10665/373900

WHO and the Jameel Arts & Health. Launch event for the new Lancet series on arts and health: [Internet]. 2023 Sep. Available from: https://www.who.int/news/item/25-09-2023-ground-breaking-research-series-on-health-benefits-of-the-arts

The Behavioural and Cultural Insights Unit at the WHO Regional Office for Europe. WHO meeting on music and motherhood: health-care innovation through the arts [Internet]. 2023 Oct 6; Copenhagen. Available from: https://www.who.int/europe/news-room/events/item/2023/10/06/default-calendar/who-meeting-on-music-and-motherhood--health-care-innovation-through-the-arts

Fancourt, D. (World Health Organisation Collaborating Centre on Arts and Health). UCL lecture on Revolutionising mental health through the arts [Internet]. Public Health Voices event series; UCL Population Health Sciences; 2023 Nov. Available from: https://www.youtube.com/watch?v=WfA-kNETGMw

Fancourt, D. Culture, Health and Wellbeing | UCLG Culture Summit 2023 in Dublin [Internet]. Culture, Health and Wellbeing | UCLG Culture Summit 2023; Dublin. Available from: https://www.youtube.com/watch?v=m60ZU49lO2o

CultureForHealth web. Digital arena for showcasing the actions and the outcomes of the EU-funded bottom-up policy development for Culture & Well-being in the EU [Internet]. Available from: https://www.cultureforhealth.eu
